# Chloridotris(3,5-dimethyl-1*H*-pyrazole-κ*N* ^2^)(formato-κ*O*)copper(II)–dichlorido­bis(3,5-dimethyl-1*H*-pyrazole-κ*N* ^2^)copper(II) (2/1)

**DOI:** 10.1107/S1600536811016461

**Published:** 2011-05-07

**Authors:** Yuliya M. Davydenko, Igor O. Fritsky, Vadim O. Pavlenko, Franc Meyer, Sebastian Dechert

**Affiliations:** a Department of Chemistry, National Taras Shevchenko University, Volodymyrska Str. 64, 01601 Kiev, Ukraine; b Institut für Anorganische Chemie, Universität Göttingen, Tammannstrasse 4, 37077 Göttingen, Germany

## Abstract

The asymmetric unit of the title compound, [Cu(CHO_2_)Cl(C_5_H_8_N_2_)_3_]_2_·[CuCl_2_(C_5_H_8_N_2_)_2_] or 2[*A*]·[*B*], contains one *A* mol­ecule and one half-molecule of *B*, located on a centre of inversion. The Cu^II^ environments in *A* and *B* are different. In *A*, the Cu^II^ atom is coordinated by three N atoms from three 3,5-dimethyl-1*H*-pyrazole (*L*) ligands, one O atom from a formate ligand and a chloride anion in an axial position [Cu—Cl = 2.4275 (7) Å] in a distorted tetra­gonal–pyramidal geometry. The Cu^II^ atom in *B* is coordinated by two N atoms from two *L* ligands and two chloride anions [Cu—Cl = 2.2524 (6) Å] in a distorted square-planar geometry. In the crystal, inter­molecular N—H⋯O hydrogen bonds link mol­ecules *A* into centrosymmetric dimers. Inter­molecular N—H⋯Cl hydrogen bonds further link these dimers with the *B* mol­ecules, forming chains propagating in [101].

## Related literature

For metal complexes with pyrazole and its derivatives, see: Trofimenko (1972) ▶; La Monica & Ardizzoia (1997 ▶); Casarin *et al.* (2005 ▶); Davydenko *et al.* (2009 ▶). For details of the bio­inorganic chemistry of copper complexes with pyrazole, see: Krämer (1999 ▶); Raptis *et al.* (1999 ▶). For applications of copper complexes with pyrazole in mol­ecular magnetism and supra­molecular chemistry, see: Krämer *et al.* (2002 ▶); Seredyuk *et al.* (2007 ▶).
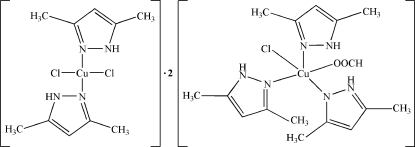


## Experimental

### 

#### Crystal data

[Cu(CHO_2_)Cl(C_5_H_8_N_2_)_3_]_2_·[CuCl_2_(C_5_H_8_N_2_)_2_]
*M* *_r_* = 1191.53
Monoclinic, 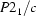

*a* = 11.4457 (3) Å
*b* = 14.4720 (5) Å
*c* = 17.0313 (5) Å
β = 106.650 (2)°
*V* = 2702.82 (14) Å^3^
*Z* = 2
Mo *K*α radiation
μ = 1.42 mm^−1^
*T* = 120 K
0.50 × 0.27 × 0.19 mm

#### Data collection

Stoe IPDS II diffractometer
Absorption correction: numerical (*X-RED32*; Stoe & Cie, 2002 ▶) *T*_min_ = 0.554, *T*_max_ = 0.763
36108 measured reflections
5749 independent reflections
4611 reflections with *I* > 2σ(*I*)
*R*_int_ = 0.031

#### Refinement

*R*[*F* ^2^ > 2σ(*F* ^2^)] = 0.034
*wR*(*F* ^2^) = 0.091
*S* = 1.01
5749 reflections
333 parameters
H atoms treated by a mixture of independent and constrained refinement
Δρ_max_ = 0.72 e Å^−3^
Δρ_min_ = −0.83 e Å^−3^

### 

Data collection: *X-AREA* (Stoe & Cie, 2002 ▶); cell refinement: *X-AREA*; data reduction: *X-AREA*; program(s) used to solve structure: *SHELXS97* (Sheldrick, 2008 ▶); program(s) used to refine structure: *SHELXL97* (Sheldrick, 2008 ▶); molecular graphics: *DIAMOND* (Brandenburg, 2001 ▶); software used to prepare material for publication: *SHELXL97*.

## Supplementary Material

Crystal structure: contains datablocks I, global. DOI: 10.1107/S1600536811016461/cv5078sup1.cif

Structure factors: contains datablocks I. DOI: 10.1107/S1600536811016461/cv5078Isup2.hkl

Additional supplementary materials:  crystallographic information; 3D view; checkCIF report

## Figures and Tables

**Table 1:** Hydrogen-bond geometry (Å, °)

*D*—H⋯*A*	*D*—H	H⋯*A*	*D*⋯*A*	*D*—H⋯*A*
N2—H2⋯Cl1	0.82 (4)	2.74 (4)	3.270 (2)	124 (3)
N2—H2⋯Cl2	0.82 (4)	2.66 (4)	3.348 (2)	143 (3)
N9—H9⋯Cl1	0.79 (4)	2.30 (4)	3.081 (2)	168 (4)
N7—H7⋯O2^i^	0.72 (4)	2.19 (4)	2.903 (3)	170 (4)
N4—H4⋯O2^i^	0.87 (4)	1.98 (4)	2.850 (3)	176 (4)
N4—H4⋯O1^i^	0.87 (4)	2.59 (4)	3.208 (3)	128 (3)

Symmetry code: (i) 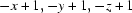.

**Fig. 1.**
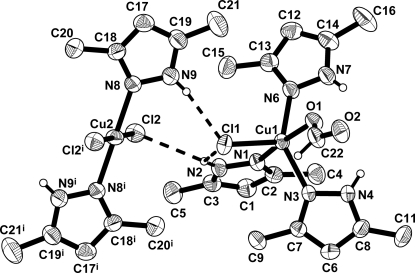
The molecular structure of (I), showing the atom-numbering scheme and the copper coordination environment [symmetry codes: (i) 2 - x, 1 - y, 2 - z]. Displacement ellipsoids are drawn at the 50% probability level. Dashed lines denote hydrogen bonds. C-bound H atoms omitted for clarity.

**Fig. 2.**
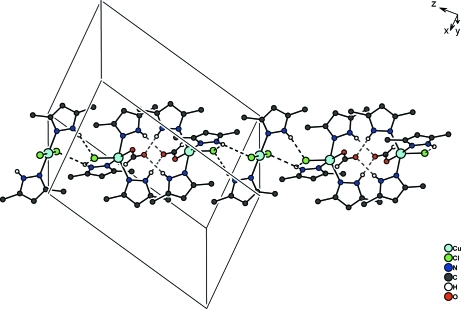
A portion of the crystal packing showing hydrogen bonds as dashed lines.

## supplementary crystallographic information

### Comment

Metal complexes with pyrazole and its derivatives have attracted much research interest for their versatile coordination chemistry and specific properties (Trofimenko, 1972; La Monica *et al.*, 1997). Pyrazole and its derivatives exhibit several coordination modes, particularly as the 1,2-bringing form, often utilized to prevent accumulation of positive charges in metal ion assembled compounds. Due to the presence of N—N bridging function in the pyrazole ring these ligands can form polynuclear complexes with specific molecular topology (Casarin *et al.*, 2005; La Monica *et al.*, 1997). In addition, neutral 1*H*-pyrazole ligands are usually bound to metal ions *via* the pyridine-type nitrogen atom thus providing formation of the mononuclear complexes (Davydenko *et al.*, 2009). Copper (II) complexes containing pyrazole-based ligands are of particular interest in bioinorganic chemistry, as they can be used as models for the active sites of copper proteins like hemocyanine and tyrosinase (Krämer, 1999; Raptis *et al.*, 1999). These compounds have been widely used in molecular magnetism as they can exhibit specific magnetic properties and in supramolecular chemistry as they can be used as building blocks for the preparation of polynuclear complexes or coordination polymers (Krämer *et al.*, 2002; Seredyuk *et al.*, 2007).

The title compound 2[*A*].[*B*], (I), reported here, contains one molecule *A* (= chloro-tris(3,5-dimethyl-1*H*-pyrazole-κ*N*)- formato-κ*O*-cooper(II)) and one-half of *B* (= dichloro-bis(3,5-dimethyl-1*H*-pyrazole-κ*N*)-cooper(II)) located on centre of inversion (Fig. 1).

In *A*, the Cu1 atom has a distorted tetragonal-pyramidal geometry with equatorial plane formed by three N atoms belonging to 3,5-dimethyl-1*H*-pyrazole ligands [Cu1—N = 1.989 (2) - 2.075 (2) Å] and one O atom from formato ligand [Cu1—O1 = 1.961 (16) Å]. The axial position is occupied by chloro anion [Cu1—Cl1 = 2.427 (7) Å]. The N—H group from one molecule of 3,5-dimethyl-1*H*-pyrazole forms an intramolecular hydrogen bond with neighboring an atom of chlorine N2–H2···Cl1 = 3.270 (2) Å. Hence, two N,H, Cl and Cu atoms form the five-membered cycle.

The Cu2 center in *B* is coordinated by two N atoms from two ligands *L* [Cu2–N = 2.014 (2) Å] and two chloro anions [Cu2—Cl 2.252 (6) Å] in a distorted square-planar geometry. The ligands of each sort are *trans*-oriented with respect to each other.

In the crystal structure (Fig. 2), intermolecular N—H···O hydrogen bonds (Table 1) link molecules *A* into centrosymmetric dimers. Intermolecular N—H···Cl hydrogen bonds link further these dimers with the molecules *B* into chains propagated in [101].

### Experimental

Compound (I) was synthesized by oxidative dissolution method at free access of air oxygen. The mixture of 3,5-dimethyl-1*H*-pyrazole (0.96 g; 0.01 mol), ammonium chloride (0.535 g; 0.01 mol) in dimethyformamide solution (15 ml) was stirred with copper powder (0.64 g; 0.01 mol) at ambient temperature until complete dissolving of solid. The resulting dark-green solution was filtered and the filtrate was left to stand at room temperature for crystallization in air. Slow evaporation of the solvent in 5 days yielded green crystals of (I) suitable for X-ray analysis.

### Refinement

N-bound H atoms were located from the difference Fourier map and refined freely with *U*_iso_ (H) fixed to 0.08. The rest H atoms were positioned geometrically and were constrained to ride on their parent atoms, with C—H = 0.93-0.96 Å and with *U*_iso_(H) fixed to 0.08.

### Crystal data

**Table:**

[Cu(CHO_2_)Cl(C_5_H_8_N_2_)_3_]_2_·[CuCl_2_(C_5_H_8_N_2_)_2_]	*F*(000) = 1234
*M**_r_* = 1191.53	*D*_x_ = 1.464 Mg m^−^^3^
Monoclinic, *P*2_1_/*c*	Mo *K*α radiation, λ = 0.71073 Å
Hall symbol: -P 2ybc	Cell parameters from 36108 reflections
*a* = 11.4457 (3) Å	θ = 1.9–26.8°
*b* = 14.4720 (5) Å	µ = 1.42 mm^−^^1^
*c* = 17.0313 (5) Å	*T* = 120 K
β = 106.650 (2)°	Block, blue
*V* = 2702.82 (14) Å^3^	0.50 × 0.27 × 0.19 mm
*Z* = 2	

### Data collection

**Table:**

Stoe IPDS II diffractometer	5749 independent reflections
Radiation source: fine-focus sealed tube	4611 reflections with *I* > 2σ(*I*)
graphite	*R*_int_ = 0.031
ω scans	θ_max_ = 26.8°, θ_min_ = 1.9°
Absorption correction: numerical (*X-RED32*; Stoe & Cie, 2002)	*h* = −14→14
*T*_min_ = 0.554, *T*_max_ = 0.763	*k* = −18→18
36108 measured reflections	*l* = −21→21

### Refinement

**Table:**

Refinement on *F*^2^	Primary atom site location: structure-invariant direct methods
Least-squares matrix: full	Secondary atom site location: difference Fourier map
*R*[*F*^2^ > 2σ(*F*^2^)] = 0.034	Hydrogen site location: inferred from neighbouring sites
*wR*(*F*^2^) = 0.091	H atoms treated by a mixture of independent and constrained refinement
*S* = 1.01	*w* = 1/[σ^2^(*F*_o_^2^) + (0.0609*P*)^2^] where *P* = (*F*_o_^2^ + 2*F*_c_^2^)/3
5749 reflections	(Δ/σ)_max_ = 0.001
333 parameters	Δρ_max_ = 0.72 e Å^−^^3^
0 restraints	Δρ_min_ = −0.83 e Å^−^^3^

### Special details

**Table:**

Geometry. All e.s.d.'s (except the e.s.d. in the dihedral angle between two l.s. planes) are estimated using the full covariance matrix. The cell e.s.d.'s are taken into account individually in the estimation of e.s.d.'s in distances, angles and torsion angles; correlations between e.s.d.'s in cell parameters are only used when they are defined by crystal symmetry. An approximate (isotropic) treatment of cell e.s.d.'s is used for estimating e.s.d.'s involving l.s. planes.
Refinement. Refinement of *F*^2^ against ALL reflections. The weighted *R*-factor *wR* and goodness of fit *S* are based on *F*^2^, conventional *R*-factors *R* are based on *F*, with *F* set to zero for negative *F*^2^. The threshold expression of *F*^2^ > σ(*F*^2^) is used only for calculating *R*-factors(gt) *etc*. and is not relevant to the choice of reflections for refinement. *R*-factors based on *F*^2^ are statistically about twice as large as those based on *F*, and *R*- factors based on ALL data will be even larger.

### Fractional atomic coordinates and isotropic or equivalent isotropic displacement parameters (Å^2^)

**Table:**

	*x*	*y*	*z*	*U*_iso_*/*U*_eq_	
Cu1	0.73059 (2)	0.426309 (19)	0.646481 (16)	0.03259 (9)	
Cu2	1.0000	0.5000	1.0000	0.03732 (11)	
Cl1	0.80389 (6)	0.51219 (4)	0.77357 (4)	0.04438 (15)	
Cl2	1.02066 (6)	0.36400 (4)	0.94106 (4)	0.04571 (15)	
N1	0.87868 (17)	0.34752 (14)	0.66725 (12)	0.0360 (4)	
N2	0.97024 (19)	0.34221 (15)	0.73835 (13)	0.0386 (4)	
N3	0.78754 (18)	0.50097 (14)	0.56033 (12)	0.0377 (4)	
N4	0.7119 (2)	0.50467 (16)	0.48203 (13)	0.0413 (5)	
N6	0.62439 (18)	0.31213 (14)	0.65350 (13)	0.0393 (4)	
N7	0.5235 (2)	0.29562 (16)	0.58991 (15)	0.0436 (5)	
N8	0.83090 (19)	0.46420 (15)	1.00220 (13)	0.0392 (4)	
N9	0.7440 (2)	0.44923 (17)	0.93053 (14)	0.0448 (5)	
C1	1.0239 (2)	0.25280 (18)	0.65202 (17)	0.0442 (6)	
H1	1.0668	0.2121	0.6284	0.080*	
C2	0.9111 (2)	0.29314 (16)	0.61396 (15)	0.0372 (5)	
C3	1.0590 (2)	0.28477 (17)	0.73078 (16)	0.0407 (5)	
C4	0.8330 (3)	0.2839 (2)	0.52783 (17)	0.0491 (6)	
H4A	0.7488	0.2820	0.5271	0.080*	
H4B	0.8535	0.2279	0.5046	0.080*	
H4C	0.8462	0.3358	0.4963	0.080*	
C5	1.1709 (3)	0.2667 (2)	0.79957 (19)	0.0563 (7)	
H5A	1.2286	0.3156	0.8024	0.080*	
H5B	1.2063	0.2090	0.7905	0.080*	
H5C	1.1499	0.2638	0.8502	0.080*	
C6	0.8677 (3)	0.5884 (2)	0.48219 (18)	0.0547 (7)	
H6	0.9216	0.6269	0.4661	0.080*	
C7	0.8829 (2)	0.55265 (17)	0.56019 (15)	0.0385 (5)	
C8	0.7589 (2)	0.55637 (19)	0.43374 (16)	0.0443 (6)	
C9	0.9877 (2)	0.5650 (2)	0.63566 (18)	0.0543 (7)	
H9A	0.9576	0.5687	0.6827	0.080*	
H9B	1.0305	0.6209	0.6311	0.080*	
H9C	1.0421	0.5133	0.6415	0.080*	
C11	0.6941 (3)	0.5685 (3)	0.34459 (17)	0.0650 (9)	
H11A	0.7164	0.5193	0.3139	0.080*	
H11B	0.7169	0.6267	0.3261	0.080*	
H11C	0.6076	0.5674	0.3365	0.080*	
C12	0.5155 (3)	0.19598 (19)	0.68291 (19)	0.0517 (7)	
H12	0.4911	0.1484	0.7114	0.080*	
C13	0.6201 (2)	0.25102 (17)	0.71056 (16)	0.0423 (6)	
C14	0.4565 (2)	0.22632 (18)	0.60563 (19)	0.0492 (6)	
C15	0.7171 (3)	0.2453 (2)	0.79031 (18)	0.0579 (7)	
H15A	0.7810	0.2052	0.7847	0.080*	
H15B	0.6830	0.2213	0.8315	0.080*	
H15C	0.7498	0.3058	0.8061	0.080*	
C16	0.3415 (3)	0.1954 (2)	0.5443 (3)	0.0763 (11)	
H16A	0.3058	0.2464	0.5097	0.080*	
H16B	0.2853	0.1733	0.5724	0.080*	
H16C	0.3597	0.1465	0.5115	0.080*	
C17	0.6545 (3)	0.4272 (2)	1.02527 (18)	0.0501 (6)	
H17	0.5969	0.4145	1.0527	0.080*	
C18	0.7763 (2)	0.45062 (17)	1.06020 (16)	0.0406 (5)	
C19	0.6368 (2)	0.4267 (2)	0.94247 (18)	0.0491 (6)	
C20	0.8437 (3)	0.4582 (2)	1.14949 (17)	0.0557 (7)	
H20A	0.9297	0.4616	1.1560	0.080*	
H20B	0.8265	0.4050	1.1780	0.080*	
H20C	0.8179	0.5130	1.1717	0.080*	
C21	0.5268 (3)	0.4061 (3)	0.8719 (2)	0.0783 (11)	
H21A	0.5397	0.4290	0.8221	0.080*	
H21B	0.4566	0.4357	0.8808	0.080*	
H21C	0.5138	0.3406	0.8676	0.080*	
C22	0.5712 (2)	0.57910 (18)	0.60437 (17)	0.0472 (6)	
H22	0.6418	0.6111	0.6312	0.080*	
O1	0.57534 (15)	0.49305 (11)	0.60884 (11)	0.0400 (4)	
O2	0.48246 (17)	0.62519 (13)	0.56778 (12)	0.0514 (5)	
H2	0.966 (3)	0.371 (3)	0.779 (2)	0.080*	
H4	0.650 (4)	0.467 (3)	0.467 (2)	0.080*	
H7	0.515 (4)	0.318 (3)	0.551 (2)	0.080*	
H9	0.753 (4)	0.459 (3)	0.887 (3)	0.080*	

### Atomic displacement parameters (Å^2^)

**Table:**

	*U*^11^	*U*^22^	*U*^33^	*U*^12^	*U*^13^	*U*^23^
Cu1	0.02773 (14)	0.03492 (15)	0.03263 (15)	0.00171 (11)	0.00467 (11)	0.00305 (11)
Cu2	0.0337 (2)	0.0406 (2)	0.0404 (2)	−0.00253 (17)	0.01504 (18)	−0.00089 (17)
Cl1	0.0514 (4)	0.0500 (3)	0.0289 (3)	0.0016 (3)	0.0069 (3)	−0.0006 (2)
Cl2	0.0451 (3)	0.0447 (3)	0.0490 (4)	−0.0002 (3)	0.0162 (3)	−0.0035 (3)
N1	0.0305 (10)	0.0389 (10)	0.0353 (10)	0.0008 (8)	0.0043 (8)	0.0000 (8)
N2	0.0339 (10)	0.0426 (11)	0.0360 (11)	0.0053 (8)	0.0048 (9)	0.0021 (9)
N3	0.0307 (10)	0.0482 (11)	0.0299 (10)	−0.0041 (8)	0.0016 (8)	0.0045 (8)
N4	0.0347 (11)	0.0536 (13)	0.0306 (10)	−0.0059 (9)	0.0015 (8)	0.0066 (9)
N6	0.0320 (10)	0.0388 (10)	0.0422 (11)	−0.0006 (8)	0.0028 (8)	0.0047 (9)
N7	0.0330 (11)	0.0440 (12)	0.0471 (13)	−0.0031 (9)	0.0009 (10)	0.0044 (10)
N8	0.0363 (11)	0.0455 (11)	0.0360 (11)	−0.0069 (9)	0.0104 (9)	−0.0047 (9)
N9	0.0426 (12)	0.0569 (13)	0.0358 (11)	−0.0059 (10)	0.0128 (10)	−0.0056 (10)
C1	0.0409 (14)	0.0433 (13)	0.0503 (15)	0.0070 (11)	0.0163 (12)	0.0002 (11)
C2	0.0360 (12)	0.0344 (11)	0.0423 (13)	−0.0010 (9)	0.0129 (10)	−0.0023 (10)
C3	0.0318 (12)	0.0401 (12)	0.0494 (14)	0.0051 (10)	0.0103 (11)	0.0093 (11)
C4	0.0466 (15)	0.0544 (15)	0.0444 (15)	−0.0014 (12)	0.0099 (12)	−0.0135 (12)
C5	0.0424 (15)	0.0629 (18)	0.0571 (18)	0.0138 (13)	0.0037 (13)	0.0100 (14)
C6	0.0412 (15)	0.075 (2)	0.0484 (15)	−0.0128 (14)	0.0142 (12)	0.0134 (14)
C7	0.0308 (12)	0.0460 (13)	0.0384 (12)	−0.0018 (10)	0.0096 (10)	−0.0004 (10)
C8	0.0412 (14)	0.0568 (16)	0.0355 (13)	0.0047 (11)	0.0118 (11)	0.0084 (11)
C9	0.0361 (14)	0.077 (2)	0.0465 (15)	−0.0117 (13)	0.0062 (12)	−0.0054 (14)
C11	0.0569 (19)	0.102 (3)	0.0348 (14)	0.0057 (17)	0.0115 (13)	0.0173 (16)
C12	0.0530 (17)	0.0415 (14)	0.0631 (18)	−0.0055 (12)	0.0205 (14)	0.0070 (13)
C13	0.0449 (14)	0.0368 (12)	0.0453 (14)	0.0025 (10)	0.0131 (11)	0.0039 (10)
C14	0.0389 (14)	0.0405 (13)	0.0656 (18)	−0.0046 (11)	0.0106 (13)	−0.0041 (13)
C15	0.066 (2)	0.0556 (17)	0.0450 (16)	−0.0010 (14)	0.0052 (14)	0.0090 (13)
C16	0.0484 (18)	0.0609 (19)	0.102 (3)	−0.0152 (15)	−0.0063 (18)	−0.0046 (19)
C17	0.0449 (15)	0.0578 (16)	0.0528 (16)	−0.0035 (12)	0.0226 (13)	−0.0044 (13)
C18	0.0435 (14)	0.0404 (12)	0.0403 (13)	−0.0034 (10)	0.0160 (11)	−0.0014 (10)
C19	0.0377 (14)	0.0598 (16)	0.0485 (15)	−0.0013 (12)	0.0103 (12)	−0.0095 (13)
C20	0.0581 (18)	0.0687 (18)	0.0406 (15)	−0.0085 (15)	0.0145 (13)	−0.0011 (13)
C21	0.0467 (18)	0.111 (3)	0.069 (2)	−0.0045 (19)	0.0029 (16)	−0.025 (2)
C22	0.0368 (13)	0.0419 (14)	0.0519 (15)	−0.0009 (11)	−0.0048 (11)	0.0017 (12)
O1	0.0300 (8)	0.0390 (9)	0.0474 (10)	0.0006 (7)	0.0055 (7)	0.0020 (7)
O2	0.0421 (11)	0.0473 (10)	0.0548 (11)	0.0111 (8)	−0.0022 (9)	0.0037 (9)

### Geometric parameters (Å, °)

**Table:**

Cu1—O1	1.9618 (16)	C6—C8	1.363 (4)
Cu1—N1	1.989 (2)	C6—C7	1.389 (4)
Cu1—N3	2.072 (2)	C6—H6	0.9300
Cu1—N6	2.075 (2)	C7—C9	1.496 (4)
Cu1—Cl1	2.4275 (7)	C8—C11	1.497 (4)
Cu2—N8^i^	2.014 (2)	C9—H9A	0.9600
Cu2—N8	2.014 (2)	C9—H9B	0.9600
Cu2—Cl2	2.2524 (6)	C9—H9C	0.9600
Cu2—Cl2^i^	2.2524 (6)	C11—H11A	0.9600
N1—C2	1.332 (3)	C11—H11B	0.9600
N1—N2	1.358 (3)	C11—H11C	0.9600
N2—C3	1.347 (3)	C12—C14	1.368 (4)
N2—H2	0.82 (4)	C12—C13	1.403 (4)
N3—C7	1.324 (3)	C12—H12	0.9300
N3—N4	1.367 (3)	C13—C15	1.490 (4)
N4—C8	1.334 (3)	C14—C16	1.495 (4)
N4—H4	0.87 (4)	C15—H15A	0.9600
N6—C13	1.325 (3)	C15—H15B	0.9600
N6—N7	1.359 (3)	C15—H15C	0.9600
N7—C14	1.336 (4)	C16—H16A	0.9600
N7—H7	0.72 (4)	C16—H16B	0.9600
N8—C18	1.326 (3)	C16—H16C	0.9600
N8—N9	1.353 (3)	C17—C19	1.366 (4)
N9—C19	1.340 (4)	C17—C18	1.392 (4)
N9—H9	0.79 (4)	C17—H17	0.9300
C1—C3	1.366 (4)	C18—C20	1.499 (4)
C1—C2	1.395 (4)	C19—C21	1.500 (4)
C1—H1	0.9300	C20—H20A	0.9600
C2—C4	1.489 (4)	C20—H20B	0.9600
C3—C5	1.491 (4)	C20—H20C	0.9600
C4—H4A	0.9600	C21—H21A	0.9600
C4—H4B	0.9600	C21—H21B	0.9600
C4—H4C	0.9600	C21—H21C	0.9600
C5—H5A	0.9600	C22—O2	1.226 (3)
C5—H5B	0.9600	C22—O1	1.248 (3)
C5—H5C	0.9600	C22—H22	0.9300
			
O1—Cu1—N1	170.57 (8)	N3—C7—C6	109.5 (2)
O1—Cu1—N3	87.20 (8)	N3—C7—C9	121.6 (2)
N1—Cu1—N3	89.99 (8)	C6—C7—C9	128.8 (2)
O1—Cu1—N6	85.42 (7)	N4—C8—C6	106.1 (2)
N1—Cu1—N6	90.96 (8)	N4—C8—C11	121.4 (3)
N3—Cu1—N6	139.85 (8)	C6—C8—C11	132.5 (3)
O1—Cu1—Cl1	95.08 (5)	C7—C9—H9A	109.5
N1—Cu1—Cl1	94.34 (6)	C7—C9—H9B	109.5
N3—Cu1—Cl1	105.43 (6)	H9A—C9—H9B	109.5
N6—Cu1—Cl1	114.51 (6)	C7—C9—H9C	109.5
N8^i^—Cu2—N8	180.0	H9A—C9—H9C	109.5
N8^i^—Cu2—Cl2	89.56 (6)	H9B—C9—H9C	109.5
N8—Cu2—Cl2	90.44 (6)	C8—C11—H11A	109.5
N8^i^—Cu2—Cl2^i^	90.44 (6)	C8—C11—H11B	109.5
N8—Cu2—Cl2^i^	89.56 (6)	H11A—C11—H11B	109.5
Cl2—Cu2—Cl2^i^	180.000 (1)	C8—C11—H11C	109.5
C2—N1—N2	106.2 (2)	H11A—C11—H11C	109.5
C2—N1—Cu1	127.41 (17)	H11B—C11—H11C	109.5
N2—N1—Cu1	126.29 (16)	C14—C12—C13	106.2 (2)
C3—N2—N1	111.1 (2)	C14—C12—H12	126.9
C3—N2—H2	128 (3)	C13—C12—H12	126.9
N1—N2—H2	120 (3)	N6—C13—C12	109.9 (2)
C7—N3—N4	105.55 (19)	N6—C13—C15	122.1 (2)
C7—N3—Cu1	136.28 (17)	C12—C13—C15	128.0 (2)
N4—N3—Cu1	118.13 (15)	N7—C14—C12	106.3 (2)
C8—N4—N3	111.7 (2)	N7—C14—C16	121.8 (3)
C8—N4—H4	127 (3)	C12—C14—C16	132.0 (3)
N3—N4—H4	120 (3)	C13—C15—H15A	109.5
C13—N6—N7	105.4 (2)	C13—C15—H15B	109.5
C13—N6—Cu1	135.97 (18)	H15A—C15—H15B	109.5
N7—N6—Cu1	118.17 (16)	C13—C15—H15C	109.5
C14—N7—N6	112.2 (2)	H15A—C15—H15C	109.5
C14—N7—H7	126 (3)	H15B—C15—H15C	109.5
N6—N7—H7	121 (3)	C14—C16—H16A	109.5
C18—N8—N9	105.5 (2)	C14—C16—H16B	109.5
C18—N8—Cu2	135.42 (18)	H16A—C16—H16B	109.5
N9—N8—Cu2	119.08 (15)	C14—C16—H16C	109.5
C19—N9—N8	111.7 (2)	H16A—C16—H16C	109.5
C19—N9—H9	124 (3)	H16B—C16—H16C	109.5
N8—N9—H9	124 (3)	C19—C17—C18	106.1 (2)
C3—C1—C2	106.7 (2)	C19—C17—H17	127.0
C3—C1—H1	126.7	C18—C17—H17	127.0
C2—C1—H1	126.7	N8—C18—C17	110.2 (2)
N1—C2—C1	109.4 (2)	N8—C18—C20	122.0 (2)
N1—C2—C4	121.2 (2)	C17—C18—C20	127.8 (2)
C1—C2—C4	129.4 (2)	N9—C19—C17	106.5 (2)
N2—C3—C1	106.6 (2)	N9—C19—C21	121.4 (3)
N2—C3—C5	122.3 (2)	C17—C19—C21	132.1 (3)
C1—C3—C5	131.1 (2)	C18—C20—H20A	109.5
C2—C4—H4A	109.5	C18—C20—H20B	109.5
C2—C4—H4B	109.5	H20A—C20—H20B	109.5
H4A—C4—H4B	109.5	C18—C20—H20C	109.5
C2—C4—H4C	109.5	H20A—C20—H20C	109.5
H4A—C4—H4C	109.5	H20B—C20—H20C	109.5
H4B—C4—H4C	109.5	C19—C21—H21A	109.5
C3—C5—H5A	109.5	C19—C21—H21B	109.5
C3—C5—H5B	109.5	H21A—C21—H21B	109.5
H5A—C5—H5B	109.5	C19—C21—H21C	109.5
C3—C5—H5C	109.5	H21A—C21—H21C	109.5
H5A—C5—H5C	109.5	H21B—C21—H21C	109.5
H5B—C5—H5C	109.5	O2—C22—O1	125.9 (2)
C8—C6—C7	107.2 (2)	O2—C22—H22	117.1
C8—C6—H6	126.4	O1—C22—H22	117.1
C7—C6—H6	126.4	C22—O1—Cu1	121.76 (16)
			
N3—Cu1—N1—C2	−61.4 (2)	C3—C1—C2—C4	178.6 (3)
N6—Cu1—N1—C2	78.5 (2)	N1—N2—C3—C1	0.6 (3)
Cl1—Cu1—N1—C2	−166.9 (2)	N1—N2—C3—C5	179.6 (2)
N3—Cu1—N1—N2	115.12 (19)	C2—C1—C3—N2	−0.4 (3)
N6—Cu1—N1—N2	−105.02 (19)	C2—C1—C3—C5	−179.2 (3)
Cl1—Cu1—N1—N2	9.65 (19)	N4—N3—C7—C6	−0.2 (3)
C2—N1—N2—C3	−0.6 (3)	Cu1—N3—C7—C6	−177.8 (2)
Cu1—N1—N2—C3	−177.71 (17)	N4—N3—C7—C9	−179.3 (2)
O1—Cu1—N3—C7	128.2 (3)	Cu1—N3—C7—C9	3.1 (4)
N1—Cu1—N3—C7	−60.8 (3)	C8—C6—C7—N3	−0.2 (3)
N6—Cu1—N3—C7	−152.3 (2)	C8—C6—C7—C9	178.7 (3)
Cl1—Cu1—N3—C7	33.7 (3)	N3—N4—C8—C6	−0.8 (3)
O1—Cu1—N3—N4	−49.16 (18)	N3—N4—C8—C11	178.3 (3)
N1—Cu1—N3—N4	121.84 (18)	C7—C6—C8—N4	0.6 (3)
N6—Cu1—N3—N4	30.4 (2)	C7—C6—C8—C11	−178.3 (3)
Cl1—Cu1—N3—N4	−143.66 (16)	N7—N6—C13—C12	−0.3 (3)
C7—N3—N4—C8	0.7 (3)	Cu1—N6—C13—C12	171.4 (2)
Cu1—N3—N4—C8	178.75 (18)	N7—N6—C13—C15	178.8 (2)
O1—Cu1—N6—C13	−125.8 (3)	Cu1—N6—C13—C15	−9.4 (4)
N1—Cu1—N6—C13	62.9 (3)	C14—C12—C13—N6	0.4 (3)
N3—Cu1—N6—C13	154.0 (2)	C14—C12—C13—C15	−178.7 (3)
Cl1—Cu1—N6—C13	−32.3 (3)	N6—N7—C14—C12	0.1 (3)
O1—Cu1—N6—N7	45.19 (18)	N6—N7—C14—C16	−179.6 (3)
N1—Cu1—N6—N7	−126.09 (19)	C13—C12—C14—N7	−0.3 (3)
N3—Cu1—N6—N7	−35.0 (2)	C13—C12—C14—C16	179.4 (3)
Cl1—Cu1—N6—N7	138.70 (17)	N9—N8—C18—C17	−0.2 (3)
C13—N6—N7—C14	0.1 (3)	Cu2—N8—C18—C17	179.3 (2)
Cu1—N6—N7—C14	−173.38 (18)	N9—N8—C18—C20	178.3 (2)
Cl2—Cu2—N8—C18	117.9 (3)	Cu2—N8—C18—C20	−2.2 (4)
Cl2^i^—Cu2—N8—C18	−62.1 (3)	C19—C17—C18—N8	0.2 (3)
Cl2—Cu2—N8—N9	−62.61 (18)	C19—C17—C18—C20	−178.1 (3)
Cl2^i^—Cu2—N8—N9	117.39 (18)	N8—N9—C19—C17	0.2 (3)
C18—N8—N9—C19	0.0 (3)	N8—N9—C19—C21	−179.5 (3)
Cu2—N8—N9—C19	−179.60 (19)	C18—C17—C19—N9	−0.2 (3)
N2—N1—C2—C1	0.3 (3)	C18—C17—C19—C21	179.4 (4)
Cu1—N1—C2—C1	177.43 (17)	O2—C22—O1—Cu1	165.4 (2)
N2—N1—C2—C4	−178.4 (2)	N3—Cu1—O1—C22	−51.9 (2)
Cu1—N1—C2—C4	−1.3 (3)	N6—Cu1—O1—C22	167.6 (2)
C3—C1—C2—N1	0.0 (3)	Cl1—Cu1—O1—C22	53.3 (2)

Symmetry codes: (i) −*x*+2, −*y*+1, −*z*+2.

### Hydrogen-bond geometry (Å, °)

**Table:**

*D*—H···*A*	*D*—H	H···*A*	*D*···*A*	*D*—H···*A*
N2—H2···Cl1	0.82 (4)	2.74 (4)	3.270 (2)	124 (3)
N2—H2···Cl2	0.82 (4)	2.66 (4)	3.348 (2)	143 (3)
N9—H9···Cl1	0.79 (4)	2.30 (4)	3.081 (2)	168 (4)
N7—H7···O2^ii^	0.72 (4)	2.19 (4)	2.903 (3)	170 (4)
N4—H4···O2^ii^	0.87 (4)	1.98 (4)	2.850 (3)	176 (4)
N4—H4···O1^ii^	0.87 (4)	2.59 (4)	3.208 (3)	128 (3)

Symmetry codes: (ii) −*x*+1, −*y*+1, −*z*+1.

## References

[bb1] Brandenburg, K. (2001). *DIAMOND* Crystal Impact GbR, Bonn, Germany.

[bb2] Casarin, M., Corvaja, C., Di Nicola, C., Falcomer, D., Franco, L., Monari, M., Pandolfo, L., Pettinari, C. & Piccinelli, F. (2005). *Inorg. Chem.* **44**, 6265–6276. 10.1021/ic050678l 16124805

[bb3] Davydenko, Y. M., Fritsky, I. O., Pavlenko, V. O., Meyer, F. & Dechert, S. (2009). *Acta Cryst.* E**65**, m691–m692. 10.1107/S1600536809019400 PMC2969789 21583048

[bb4] Krämer, R. (1999). *Coord. Chem. Rev.* **182**, 211–243.

[bb5] Krämer, R., Fritsky, I. O., Pritzkow, H. & Kowbasyuk, L. A. (2002). *J. Chem. Soc. Dalton Trans.* pp. 1307–1314.

[bb6] La Monica, G. & Ardizzoia, G. A. (1997). *Prog. Inorg. Chem.* **46**, 151–238.

[bb7] Raptis, R., Georgakaki, I. & Hockless, D. (1999). *Angew. Chem. Int. Ed.* **38**, 1632–1634. 10.1002/(SICI)1521-3773(19990601)38:11<1632::AID-ANIE1632>3.0.CO;2-O 29710973

[bb8] Seredyuk, M., Haukka, M., Fritsky, I. O., Kozlowski, H., Krämer, R., Pavlenko, V. A. & Gütlich, P. (2007). *Dalton Trans.* pp. 3183–3194. 10.1039/b702574b 17637993

[bb9] Sheldrick, G. M. (2008). *Acta Cryst.* A**64**, 112–122. 10.1107/S0108767307043930 18156677

[bb10] Stoe & Cie (2002). *X-AREA* and *X-RED32* Stoe & Cie, Darmstadt, Germany.

[bb11] Trofimenko, S. (1972). *Chem. Rev.* **93**, 943–980.

